# Contribution of *Anopheles gambiae *sensu lato mosquitoes to malaria transmission during the dry season in Djoumouna and Ntoula villages in the Republic of the Congo

**DOI:** 10.1186/s13071-023-06102-7

**Published:** 2024-03-02

**Authors:** Jacques Dollon Mbama Ntabi, Espoir Divin Malda Bali, Abel Lissom, Romaric Akoton, Jean Claude Djontu, Georges Missontsa, Freisnel Hermeland Mouzinga, Marcel Tapsou Baina, Luc Djogbenou, Cyrille Ndo, Charles Wondji, Ayola Akim Adegnika, Arsène Lenga, Steffen Borrmann, Francine Ntoumi

**Affiliations:** 1 Fondation Congolaise Pour La Recherche Médicale, Brazzaville, Republic of the Congo; 2 Faculté des Sciences et Techniques, Université Marien Ngouabi, Brazzaville, Republic of the Congo; 3 Institute of Tropical Medicine, University of Tübingen, Tübingen, Germany; 4 Department of Biological Sciences, Faculty of Science, University of Bamenda, Bamenda, Cameroon; 5 Fondation Pour La Recherche Scientifique (FORS), ISBA, BP: 88, Cotonou, Bénin; 6 Centre de Recherche Médicale de Lambaréné, Lambaréné, Gabon; 7 German Center of Infection Research (DZIF), Tübingen, Germany; 8 Department of Parasitology and Medical Entomology, Center for Research in Infectious Diseases (CRID), Yaoundé, Cameroon; 9 Department of Vector Biology, Liverpool School of Tropical Medicine (LSTM), Liverpool, United Kingdom; 10 Tropical Infectious Diseases Research Center (TIDRC), University of Abomey-Calavi, Cotonou, Bénin; 11 Department of Biological Sciences, Faculty of Medicine and Pharmaceutical Sciences, University of Douala, Douala, Cameroon; 12 Department of Parasitology and Microbiology, Center for Research in Infectious Diseases (CRID), Yaoundé, Cameroon

**Keywords:** *Anopheles gambiae *sensu lato, *Plasmodium species*, Malaria transmission, Rural areas, Republic of the Congo

## Abstract

**Background:**

Mosquitoes belonging to the *Anopheles gambiae* sensu lato complex play a major role in malaria transmission across Africa. This study assessed the relative importance of members of *An. gambiae* s.l. in malaria transmission in two rural villages in the Republic of the Congo.

**Methods:**

Adult mosquitoes were collected using electric aspirators from June to September 2022 in Djoumouna and Ntoula villages and were sorted by taxa based on their morphological features. *Anopheles gambiae* s.l. females were also molecularly identified. A TaqMan-based assay and a nested polymerase chain reaction (PCR) were performed to determine *Plasmodium* spp. in the mosquitoes. Entomological indexes were estimated, including man-biting rate, entomological inoculation rate (EIR), and diversity index.

**Results:**

Among 176 mosquitoes collected, *An. gambiae* s.l. was predominant (85.8%), followed by *Culex* spp. (13.6%) and *Aedes* spp. (0.6%). Three members of the *An. gambiae* s.l. complex were collected in both villages, namely *An. gambiae* sensu stricto (74.3%), *Anopheles coluzzii* (22.9%) and *Anopheles arabiensis* (2.8%). Three *Plasmodium* species were detected in *An. gambiae* s.s. and *An. coluzzii* (*Plasmodium falciparum, P. malariae* and *P. ovale*), while only *P. falciparum* and *P. malariae* were found in *An. arabiensis*. In general, the *Plasmodium* infection rate was 35.1% (53/151) using the TaqMan-based assay, and nested PCR confirmed 77.4% (41/53) of those infections. The nightly EIR of *An. gambiae* s.l. was 0.125 infectious bites per person per night (ib/p/n) in Djoumouna and 0.08 ib/p/n in Ntoula. The EIR of *An. gambiae* s.s. in Djoumouna (0.11 ib/p/n) and Ntoula (0.04 ib/p/n) was higher than that of *An. coluzzii* (0.01 and 0.03 ib/p/n) and *An. arabiensis* (0.005 and 0.0 ib/p/n).

**Conclusions:**

This study provides baseline information on the dominant vectors and dynamics of malaria transmission in the rural areas of the Republic of the Congo during the dry season. In the two sampled villages, *An. gambiae* s.s. appears to play a predominant role in *Plasmodium* spp. transmission.

**Graphical Abstract:**

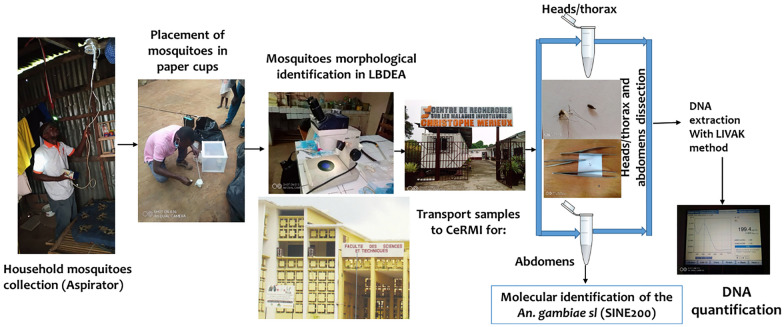

**Supplementary Information:**

The online version contains supplementary material available at 10.1186/s13071-023-06102-7.

## Background

Despite the efforts deployed against malaria in the last decade, the disease remains a significant public health problem worldwide, with an estimated 247 million cases and 619,000 deaths occurring globally in 2021 [1]. The World Health Organization African Region (WHO Africa) is still the hardest hit by malaria, accounting for 95% of cases and 96% of deaths [1]. In the Republic of the Congo, the estimated number of malaria cases reported in 2021 was 146,262, and the disease was the cause of 63% of medical consultations, 20% of hospitalisations and 9% of individual deaths in the country [2].

Malaria is transmitted through the bite of an infected female *Anopheles* mosquito, and studies conducted in the Republic of the Congo have reported species of the *Anopheles gambiae* sensu lato (s.l.) complex as major malaria vectors [2–4]. *Anopheles gambiae* s.l. is the most effective vector in the Afrotropical realm due to its high abundance, longevity, high propensity for feeding and high vectorial capacity [5–8].

*Anopheles gambiae *s.l. is a group of nine species that are indistinguishable morphologically but differ in genetic characteristics [6, 8]. They include *An. gambiae* sensu stricto (s.s.), *An. coluzzii*, *An. arabiensis*, *An. melas*, *An. merus*, *An. bwambae*, *An. quadriannulatus*, *An. amharicus* and *An. fontenillei* [8, 9]. These species are distributed mainly in sub-Saharan Africa and are capable of living in different environmental conditions [5, 10–13].

Five species of *Plasmodium* have been well described as the pathogens of malaria in humans: *Plasmodium falciparum* (the most prevalent species), *P. malariae*, *P. ovale*, *P. vivax* and *P. knowlesi* [13, 14]. While *An. gambiae* s.s., *An. coluzzii* and *An. arabiensis* are significant malaria vectors for *Plasmodium* spp. in Africa [8, 9, 11], other species including *An. melas*, *An. merus*, *An. bwambae*, *An. quadriannulatus* and *An. fontenillei* may be important in malaria transmission in specific localities [8, 9].

It is well known that malaria transmission is much higher in the rainy season than the dry season [11, 15], and some members of the *An. gambiae* s.l. complex disappear entirely during the long dry season and reappear in large numbers with the first rains [16, 17]. Even with a low transmission period (dry season), malaria continues to pose a major public health threat to communities [16]. Detailed knowledge of the vectors is necessary to identify effective control measures against local strains and populations of these vectors. Due to the complexity of the vector system, the precise identification of vectors of the complex/group of malaria in each area using a molecular tool is important for achieving better adapted, targeted and effective vector control.

Previous studies in the Republic of the Congo have reported the predominance of *An. gambiae* s.l. complex in the rural area of Djoumouna using morphological analysis, which does not allow for discrimination of its different species [18, 19]. To close this gap, this study aimed to investigate the diversity of *An. gambiae* s.l. mosquitoes and investigate their *Plasmodium* spp. infection rates during the dry season in two villages (Djoumouna and Ntoula) in the district of Goma Tsé-Tsé, in the Republic of the Congo.

## Methods

### Study area

The study was conducted in Ntoula and Djoumouna villages in the Goma Tsé-Tsé health district in the Republic of the Congo [20]. The region has a humid tropical climate with distinct seasons: dry (June to September and January to February) and rainy (October to January and March to May). Annual rainfall ranges from 1600 to 2000 mm. Temperatures average 20–32 °C, with humidity between 78 and 84% [20]. Djoumouna, 25 km from Brazzaville, is surrounded by four rivers (Lomba, Kinkoue, Loumbangala, and Djoumouna) feeding into fish ponds, potential malaria vector sites [4]. Ntoula, 30 km southeast of Brazzaville, has several rivers (Congo, Ntoula, Loumou), promoting the development of *Anopheles* mosquito larvae [19]. The primary occupations of the inhabitants of both villages are farming and fishing.

### Mosquito collection and processing

The study was conducted during the dry season. Mosquito collection was carried out for four consecutive months from June to September 2022. Mosquitoes were collected indoors between 5:00 and 10:00 from 8–10 houses each week using an electric aspirator (Rule In-Line Blowers, Model 240, China). A total of 90 houses in which at least one *Anopheles* was collected (52 houses in Djoumouna and 38 houses in Ntoula) were included in the study. After collection, mosquitoes were kept in a small paper cup and transported to the laboratory.

Information including the number of collected mosquitoes, type of house, type of walls, number of rooms per house, number of people living in the home, number of people sleeping under the nets, date and time of collection and number of animals was recorded on the collection sheet (questionnaire). Once collected, anophelines were separated from culicines, and anopheline species were identified morphologically [21]. Each anopheline female was recorded according to the physiological status of the abdomen (unfed, blood-fed, semi-gravid, gravid). Ovarian dissection was performed on *An. gambiae* s.l. females with an empty stomach (unfed), as described previously by Champ et al. [22]. Mosquitoes were then individually stored in a well-labelled tube containing desiccant and kept in a freezer at −20 °C for subsequent analysis.

### Molecular identification of *Anopheles* species

Whole mosquito DNA was extracted from 144 female mosquitoes (*An. gambiae* complex) using the Livak extraction method [23]. Extracted DNA samples were subjected to polymerase chain reaction (PCR) analysis [24] using SINE200 primers which target retrotransposons of *An. gambiae* s.l. species, thereby allowing us to distinguish *An. coluzzii* from *An. gambiae* s.s. and *An. arabiensis* [24]. The PCR mix was carried out in 14 µl reaction volume of master mix (1.5 µl of PCR buffer 10×, 0.75 µl of 25 mM MgCl_2_, 0.12 µl of 10 mM dNTPs, 0.51 µl of 10 µM SINE_Foward and 0.51 µl of 10 µM SINE_Reverse primers, 0.12 µl of KAPA *Taq* DNA polymerase 5U/µl, and 10.49 µl of nuclease-free water). A volume of 1 μl of genomic DNA was added to the master mix as a template, and the amplification was performed in a thermal cycler (Mastercycler X50a, Eppendorf AG, Hamburg, Germany) using initial denaturation at 95 °C for 5 min, followed by 35 cycles of denaturation at 95 °C for 30 s, annealing at 54 °C for 1 min and extension at 72 °C for 1 min, with a final extension at 72 °C for 10 min and hold at 10 °C. The details of primers targeting SINE200 retrotransposons of *An. gambiae* s.l. are presented in Additional file 1: Table S1. PCR products and the 100-base-pair (bp) molecular weight marker were stained with SYBR Green solution (1:1, v/v), electrophoresed at 100 V for 50 min in a 1.5% agarose gel and visualised on a GelDoc™ EZ Imager (Bio-Rad Laboratories, Hercules, CA, USA). A sample was considered positive for *An. gambiae*, *An. coluzzii* or *An. arabiensis* if a 249-bp, 479-bp or 223-bp band was detected, respectively.

### Detection of *Plasmodium* spp. in *An. gambiae* s.l.

A TaqMan assay was used to detect the *Plasmodium* species in females of some species of *An. gambiae* s.l. This very sensitive method enables the detection of *P. falciparum* but cannot differentiate *P. malariae* from *P. ovale* and *P. vivax*. Briefly, the amplification was performed in a reaction volume of 10 µl comprising 1 µl of matrix DNA, 5 µl (1 µM) of SensiMix II Probe (1.25 ml), 0.8 µl (10 mM) of PlasF (forward primer), 0.8 µl (10 mM) of PlasR (reverse primer), 0.3 µl of Falcip+, 0.2 µl of OVM+ and 1.9 µl of nuclease-free water. The samples were amplified in a LightCycler 480 real-time PCR system (Roche, SN: 20726) using the following conditions: pre-denaturation at 95 °C for 10 min, followed by 40 cycles of 15 s at 92 °C and 1 min at 60 °C. The primers (Falcip+ and Plas-F) were used together with two probes tagged with fluorophores (FAM for the detection of *P. falciparum* and HEX to detect *P. ovale*, *P. malariae* and *P. vivax*). Two *P. falciparum* samples and a mix of *P. ovale*, *P. vivax* and *P. malariae* were used as positive controls. Details of the primers targeting the 18S ribosomal RNA (rRNA) gene of *Plasmodium* spp. and probes are provided in Additional file 1: Table S1. TaqMan-positive samples were subjected to nested PCR to confirm and discriminate *P. malariae* from *P. ovale* and *P. vivax* [25].

The first round of the nested PCR reaction consisted of selectively amplifying the DNA of the genus *Plasmodium.* This first-round PCR was carried out in a reaction volume of 20 μl consisting of PCR buffer 10×, 10 nM dNTPs, 25 mM MgCl2, 5U/µl DreamTaq DNA polymerase, distilled water, 10 µM rPLU5 forward and 10 µM rPLU6 reverse primers, and 2 µl of genomic DNA. The amplification was performed in a thermal cycler (Mastercycler X50a, Eppendorf AG, Hamburg, Germany) using an initial denaturation at 94 °C for 4 min, followed by 35 cycles of denaturation at 94 °C for 30 s, annealing at 55 °C for 1 min and extension at 72 °C for 1 min, with a final extension at 72 °C for 4 min. The second-round PCR reaction was intended for the speciation of the malaria parasite using the product of the first-round PCR reaction as a template and the primers designed to amplify the specific sequences of *P. falciparum* (rFAL1/rFL2), *P. ovale* (rOVA1/rOVA2), *P. malariae* (rMAL1/rMAL2) and *P. vivax* (rVAV1/rVAV2) as presented in Additional file 1: Table S1. For this second-round PCR reaction, 1 μl of the product of the first-round PCR was added in 19 μl of master mix prepared as described above and amplified using the thermal cycler under the same cycling conditions as described for the first-round PCR reaction, except that the annealing temperature was 58 °C. Details of the primers used are provided in the Additional file 1: Table S1.

PCR products and the 100-bp molecular weight marker were stained with SYBR Green solution (1:1, v/v), electrophoresed at 100 V for 45 min in a 1.5% agarose gel and visualised on the GelDoc™ EZ Imager (Bio-Rad Laboratories, Hercules, CA, USA). A sample was considered positive for *P. falciparum*, *P. malaria*, *P. ovale* and *P. viva*x if a 205-bp, 144-bp, 800-bp or 120-bp band was detected. The known *Plasmodium*-positive samples from our library and distilled water served as positive and negative controls in every set of reactions.

### Data analysis

All statistical tests were performed using GraphPad Prism 6.01 software. Categorical variables were represented in proportions. Fisher’s exact test was used to compare proportions of species of *An. gambiae* complex and their contribution to the transmission of *Plasmodium* species in Ntoula village versus Djoumouna. The Chi-square test was used to compare mosquito species abundance in Djoumouna and Ntoula or to compare the parity rate of the mosquitoes between the two settings. The significance threshold was set at *P* < 0.05.

The entomological indexes of malaria transmission included in this study are the man-biting rate, infection rate, entomological inoculation rate (EIR), parity rate, and resting *Anopheles* density.

The man-biting rate (ma), also called aggressive density, is the product of anopheline density in contact with humans (m) and the anthropophilia rate (a). It is calculated by dividing the total number of engorged females (F) of a species captured by the total number of people (W) who spent the night in the rooms where the captures occurred. The man-biting rate is expressed as the number of *Anopheles* mosquito bites per person per night.

$${\varvec{m}}{\varvec{a}}={\varvec{F}}\div {\varvec{W}}$$

The *Plasmodium* infection rate (s) is the proportion of mosquitoes infected or carrying sporozoites in their salivary glands. This index is expressed as a percentage (number of infected mosquitoes out of the number of mosquitoes examined × 100).

$${\varvec{s}}=\frac{{\varvec{N}}{\varvec{u}}{\varvec{m}}{\varvec{b}}{\varvec{e}}{\varvec{r}}\,\boldsymbol{ }{\varvec{o}}{\varvec{f}}\,\boldsymbol{ }{\varvec{m}}{\varvec{o}}{\varvec{s}}{\varvec{q}}{\varvec{u}}{\varvec{i}}{\varvec{t}}{\varvec{o}}{\varvec{e}}{\varvec{s}}\,\boldsymbol{ }{\varvec{p}}{\varvec{o}}{\varvec{s}}{\varvec{i}}{\varvec{t}}{\varvec{i}}{\varvec{v}}{\varvec{e}}}{{\varvec{N}}{\varvec{u}}{\varvec{m}}{\varvec{b}}{\varvec{e}}{\varvec{r}}\boldsymbol{ }{\varvec{o}}{\varvec{f}}\,\boldsymbol{ }{\varvec{m}}{\varvec{o}}{\varvec{s}}{\varvec{q}}{\varvec{u}}{\varvec{i}}{\varvec{t}}{\varvec{o}}{\varvec{e}}{\varvec{s}}\,\boldsymbol{ }{\varvec{t}}{\varvec{e}}{\varvec{s}}{\varvec{t}}{\varvec{e}}{\varvec{d}}}\boldsymbol{ }{\varvec{X}}\boldsymbol{ }100$$

The EIR is the number of infectious bites from *Anopheles* during a given period. It is expressed as infectious bites per person per night/day/week/month or year. ${\varvec{E}}{\varvec{I}}{\varvec{R}}=\left[\mathbf{M}\mathbf{a}\mathbf{n}-\mathbf{b}\mathbf{i}\mathbf{t}\mathbf{i}\mathbf{n}\mathbf{g}\,\mathbf{r}\mathbf{a}\mathbf{t}\mathbf{e}\,\left(\mathbf{m}\mathbf{a}\right)\right]\mathbf{x}\left[\mathbf{s}\mathbf{p}\mathbf{o}\mathbf{r}\mathbf{o}\mathbf{z}\mathbf{o}\mathbf{i}\mathbf{t}\mathbf{e}\,\mathbf{r}\mathbf{a}\mathbf{t}\mathbf{e}\left(\mathbf{s}\right)\right]={\varvec{m}}{\varvec{a}}\boldsymbol{ }\mathbf{x}{\varvec{s}}$

The density of resting *Anopheles* (D) is the number of resting mosquitoes inside households distributed over the number of houses surveyed and the number of nights of capture. It is expressed as the number of female mosquitoes per house per night.

$${\varvec{D}}=(\frac{{\varvec{n}}{\varvec{u}}{\varvec{m}}{\varvec{b}}{\varvec{e}}{\varvec{r}}\,\boldsymbol{ }{\varvec{o}}{\varvec{f}}\,\boldsymbol{ }{\varvec{f}}{\varvec{e}}{\varvec{m}}{\varvec{a}}{\varvec{l}}{\varvec{e}}{\varvec{s}}}{{\varvec{n}}{\varvec{u}}{\varvec{m}}{\varvec{b}}{\varvec{e}}{\varvec{r}}\,\boldsymbol{ }{\varvec{o}}{\varvec{f}}\,\boldsymbol{ }{\varvec{h}}{\varvec{o}}{\varvec{u}}{\varvec{s}}{\varvec{e}}\,\boldsymbol{ }{\varvec{p}}{\varvec{r}}{\varvec{o}}{\varvec{t}}{\varvec{e}}{\varvec{c}}{\varvec{t}}{\varvec{e}}{\varvec{d}}\boldsymbol{ }})/{\varvec{N}}{\varvec{u}}{\varvec{m}}{\varvec{b}}{\varvec{e}}{\varvec{r}}\,\boldsymbol{ }{\varvec{o}}{\varvec{f}}\,\boldsymbol{ }{\varvec{n}}{\varvec{i}}{\varvec{g}}{\varvec{h}}{\varvec{t}}{\varvec{s}}$$

The parity rate (P) is the proportion of parous females (females having spawned at least once) divided by the total number of mounted *Anopheles* (dissected). Older mosquito populations will show higher parity rates. Older populations are more likely to transmit malaria because they have lived long enough for the parasite to develop.

$${\varvec{P}}=\frac{{\varvec{n}}{\varvec{u}}{\varvec{m}}{\varvec{b}}{\varvec{e}}{\varvec{r}}\,\boldsymbol{ }{\varvec{o}}{\varvec{f}}\,\boldsymbol{ }{\varvec{p}}{\varvec{a}}{\varvec{r}}{\varvec{o}}{\varvec{u}}{\varvec{s}}\,\boldsymbol{ }{\varvec{A}}{\varvec{n}}{\varvec{o}}{\varvec{p}}{\varvec{h}}{\varvec{e}}{\varvec{l}}{\varvec{e}}{\varvec{s}}}{{\varvec{T}}{\varvec{o}}{\varvec{t}}{\varvec{a}}{\varvec{l}}\,\boldsymbol{ }{\varvec{n}}{\varvec{u}}{\varvec{m}}{\varvec{b}}{\varvec{e}}{\varvec{r}}\,\boldsymbol{ }{\varvec{o}}{\varvec{f}}\,\boldsymbol{ }{\varvec{A}}{\varvec{n}}{\varvec{o}}{\varvec{p}}{\varvec{h}}{\varvec{e}}{\varvec{l}}{\varvec{e}}{\varvec{s}}\,\boldsymbol{ }{\varvec{d}}{\varvec{i}}{\varvec{s}}{\varvec{s}}{\varvec{e}}{\varvec{c}}{\varvec{t}}{\varvec{e}}{\varvec{d}}}\mathbf{X}100$$

### Diversity index

The Shannon–Weaver (H′) and Simpson (D) diversity index were also determined in order to evaluate the diversity of *An. gambiae* complex within each surveyed site. These indexes consider either the number of anopheline species or the distribution of individuals within these species [26].

The Shannon–Weaver index (H) was developed within the framework of information theory, which assumes that the diversity of species can be measured as the information contained in a code or a message [27] to determine the diversity of species in a given environment. The Shannon index has no unit and is calculated from the following formula:

$$H=-\sum\nolimits_{n=1}^{s}{{\text{log}}}_{2}ni/N$$

where **H** is the Shannon–Weaver index, **N** is the total number, **ni** is the frequency of the species in the sampled area and **S** is the total number of species present in the sampled area.

This index varies between 0 and 5; a value close to 0 indicates very low diversity.

Simpson’s index (D) measures the probability that two randomly chosen individuals do not belong to the same species. It is inversely proportional to diversity. This formula was used to establish an index directly representative of heterogeneity by subtracting the Simpson index from its maximum value, which is 1 [26]. For an infinite sample, the index is given by the following formula:

$${\mathbf{D}}\, = \,\frac{{\sum {{\text{n}}\,\left( {{\text{n}}\, - \,1} \right)} }}{{{\text{N}}\,\left( {{\text{N}}\, - \,1} \right)}}$$

where **D** is the Simpson index, **n** is the number of individuals of a species and **N** is the total number of species captured. This index varies between 0 and 1; a value of 1 indicates a 100% chance of encountering the same species within a sample.

## Results

### Mosquito species composition

A total of 176 mosquitoes were collected in the Ntoula and Djoumouna villages. In both villages, *An. gambiae* s.l. was the most abundant species (85.8%, 151/176), followed by *Culex* spp. (13.6%, 24/176) and *Aedes* spp. (0.6%, 1/176) (*χ*^2^ = 333.7, degrees of freedom [*df*] = 2, *P* < 0.0001). In addition, 86.6% (97/112) and 84.4% (54/64) *An. gambiae* s.l. were recorded in Djoumouna and Ntoula, respectively (Table 1). The presence of *Culex* spp. was similar in Ntoula (14.1%, 9/64) and Djoumouna (13.4%, 15/112) (*P* = 0.99, odds ratio [OR] = 1.06, 95% confidence interval [CI] 0.44–2.58). *Aedes* spp. were only observed in Ntoula village, at 1.5% (1/64) frequency (Table 1).

**Table 1:** Mosquito species composition between Djoumouna and Ntoula, Goma Tsé-Tsé district

Mosquito species	Study sites		
	Djoumouna	Ntoula	*P*-value^a^
All mosquitoes % (*n*)			
*An. gambiae* s.l.	86.6 (97)	84.4 (54)	0.823
*Culex* spp.	13.4 (15)	14.1 (9)	0.99
*Aedes* spp.	0.0 (0)	1.5 (1)	NA
*An. gambiae* s.l. species % (*n*)			
*An. gambiae* s.s.	81.7 (76)	60.8 (31)	0.009
*An. coluzzii*	16.1 (15)	35.3 (18)	0.014
*An. arabiensis*	2.2 (2)	3.9 (2)	0.61

^a^The Chi-square test was used to compare proportions between groups with sample size larger than 4 (*n* ≥ 5); otherwise, Fisher’s exact test was used

### Distribution of *An. gambiae* s.l. species in study sites

Following the molecular identification of *An. gambiae* s.l. mosquitoes, three species including *An. gambiae*, *An. coluzzii* and *An. arabiensis* were found in both Djoumouna and Ntoula. Among these species, *An. gambiae* s.s. was the predominant species in Djoumouna (81.7%, 76/93) versus *An. coluzzii* (16.1%, 15/93) and *An. arabiensis* (2.2%, 2/93) (*χ*^2^ = 151.1, *df* = 2, *P* < 0.0001). Similar results were obtained in Ntoula (*An. gambiae* s.s. 60.8% [31/51], *An. coluzzii* 35.3% [18/51] and *An. arabiensis* 3.9% [2/5]) (*χ*^2^ = 37.2, *df* = 2, *P* < 0.0001). In addition, *An. gambiae* s.s. was more abundant in Djoumouna (81.7%, 76/93) than in Ntoula (60.8, 31/51) (*P* = 0.009, OR = 2.88, 95% CI 1.34–6.12), while the converse was observed with *An. coluzzii*, reported at 35.3% (18/51) in Ntoula and 16.1% (15/93) in Djoumouna (*P* = 0.014, OR = 2.84, 95% CI 1.24–6.14). No significant difference in terms of *An. arabiensis* (*P* = 0.61, OR = 0.53, 95% CI 0.08–3.53) was found between the two settings (2.2%, 2/93 in Djoumouna and 3.9%, 2/51 in Ntoula) (Table 1). A total of seven mosquitoes out of the 151 *An. gambiae* s.l. analysed were not discriminated; thus, they could belong to other species of *An. gambiae* complex.

### Diversity of *An. gambiae* complex

Two diversity indexes (Shannon–Weaver and Simpson) were used to assess the specific richness within each species of *An. gambiae* s.l. in the two surveyed villages. Shannon index data revealed that the Ntoula site had a higher diversity index score (1.15) than Djoumouna (0.78). However, according to the Simpson index, the probability of encountering *An. gambiae* s.l. was higher in Djoumouna (0.58) than in Ntoula (0.39).

### *Plasmodium* infection rates

Out of 151 *An. gambiae* s.l. analysed by TaqMan assay, 35.1% (53/151) were infected with *Plasmodium* spp. in both villages. *Plasmodium* spp. infection rates were 41.3% (40/97) and 24.1% (13/54) in Djoumouna and Ntoula, respectively. Overall, *An. gambiae* s.l. mosquitoes were mainly infected with *P. falciparum* (19.2%, 29/151) followed by *OVM*^+^ (non-*P. falciparum* species: *P. ovale*, *P. vivax* or *P. malariae*) at 7.9% (12/151) and co-infection with *P. falciparum* with at least one of the non-*P. falciparum* species at 7.9% (12/151).

**Fig. 1:**
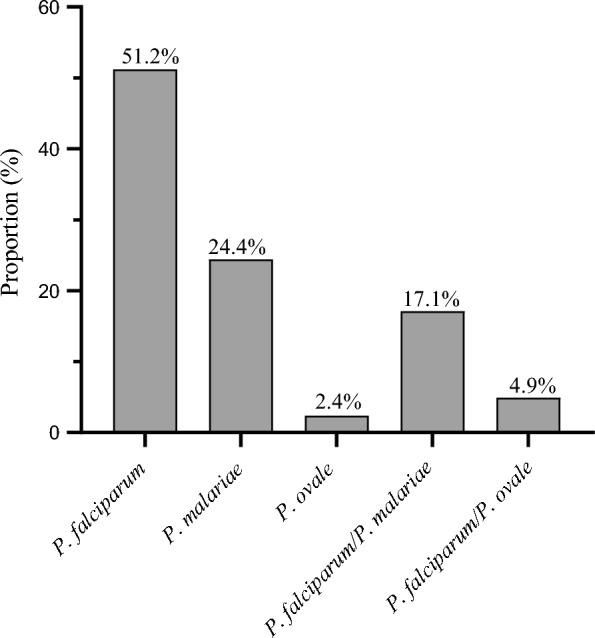
Discrimination of *Plasmodium* infection in *An. gambiae* s.l.

In this study, three *Plasmodium* species were detected in *An. gambiae* s.l.: *P. falciparum*, *P. malariae* and *P. ovale*. Among mosquito specimens testing positive by TaqMan, the nested PCR assay confirmed that 77.4% (41/53) were infected, with 51.2% (21/41) of the specimens being infected with *P. falciparum*, 24.4% (10/41) with *P. malariae*, 2.4% (1/41) with *P. ovale*, 17.1% (7/41) with *P. falciparum/P. malariae* and 4.9% (2/414) with *P. falciparum/P. ovale* (Fig. 1). In Djoumouna, 29 *An. gambiae* s.l. were infected, with 62.1%, 24.1%, 3.5%, 3.5% and 6.9% of them harbouring *P. falciparum*, *P. malariae*, *P. ovale*, *P. falciparum*/*P. malariae* and *P. falciparum*/*P. ovale*, respectively. In Ntoula, 12 *An. gambiae* s.l. were infected with *Plasmodium* spp., with 25%, 25% and 50% of them harbouring *P. falciparum*, *P. malariae* and *P. falciparum*/*P. malariae*, respectively (Table 2).

**Table 2:** Infectivity of *An. gambiae* s.l. from Djoumouna and Ntoula using nested PCR

*Plasmodium* species	Djoumouna (*n* = 29)	Ntoula (*n* = 12)	Total (*n* = 41)
*P. falciparum*	62.1 (18)	25.0 (3)	51.2 (21)
*P. malariae*	24.1 (7)	25 .0 (3)	24.4 (10)
*P. ovale*	3.5 (1)	0.0 (0)	2.4 (1)
*P. falciparum/P. malariae*	3.5 (1)	50.0 (6)	17.1 (7)
*P. falciparum/P. ovale*	6.9 (2)	0.0 (0)	4.9 (2)
Total infection rate	70.7 (29)	29.3(12)	100 (41)

**Fig. 2:**
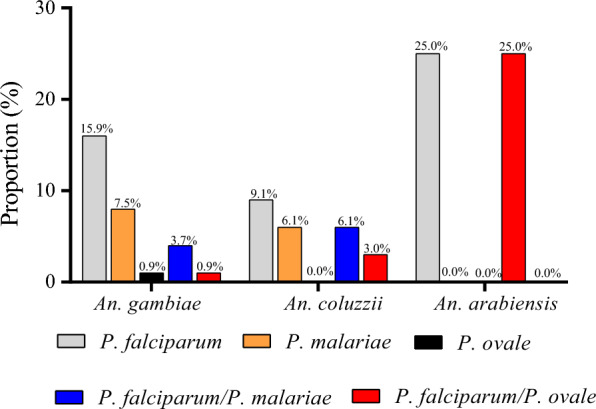
Distribution of *Plasmodium* spp. in *An. gambiae* s.l.

Overall, the *Plasmodium* spp. infection rate was higher in *An. arabiensis* (50%, 2/4) than in *An. gambiae* s.s. (20%, 31/107) and *An. coluzzii* (24.2%, 8/33), although the difference was not statistically significant (*χ*^2^ = 1.2, *df* = 2, *P* = 0.54). Moreover, there was no significant difference in terms of *Plasmodium* spp. infection rate between *An. arabiensis* (50%, 1/2), *An. gambiae* s.s. (32.9%, 25/76) and *An. coluzzii* (20%, 3/15) in Djoumouna (*χ*^2^ = 1.3, *df* = 2, *P* = 0.52). A similar trend was found in Ntoula (*An. arabiensis* [50%, 1/2], *An. coluzzii* [27.8%, 5/18] and *An. gambiae* s.s. [19.4%, 6/31]) (*χ*^2^ = 1.2, *df* = 2, *P* = 0.53) (Table 3). The proportion of mono-infection in *An. gambiae* was 15.9% for *P. falciparum*, 7.5% for *P. malariae* and 0.9% for *P. ovale*. The co-infection in *An. gambiae* s.s. was 3.7% and 0.9% from *P. falciparum*/*P. malariae* and *P. falciparum*/*P. ovale*, respectively (Fig. 2). Moreover, the proportion of infection among *An. coluzzii* was 9.1% and 6.1% for mono-infection with *P. falciparum* and *P. malariae*, respectively. The co-infection cases included 6.1% and 3.0% for *P. falciparum*/*P. malariae* and *P. falciparum*/*P. ovale*, respectively. The infection rate in *An. arabiensis* was 25% for both *P. falciparum* mono-infection and *P. falciparum*/*P. malariae* co-infection (Fig. 2). *Plasmodium falciparum* was the most prevalent *Plasmodium* spp. in all *An. gambiae* s.l. sibling species.

**Table 3:** Entomological indexes of *An. gambiae* s.l. mosquitoes

Sites	Species	Density of resting *An. gambiae* s.l. (D)	Entomological inoculation rate (EIR)	Infection rate (s)	Aggressivity rate (ma)	Parity rate (P)
Djoumouna	*An. gambiae*	0.79	0.11	0.32	0.35	85.7
	*An. coluzzii*	0.16	0.01	0.2	0.07	50
	*An. arabiensis*	0.02	0.005	0.5	0.01	0
Ntoula	*An. gambiae*	0.49	0.04	0.19	0.20	0
	*An. coluzzii*	0.20	0.03	0.27	0.10	75
	*An. arabiensis*	0.03	0.01	0.5	0.02	0

### Density of resting *Anopheles* mosquitoes

Data generated from this study revealed relatively high resting density in Djoumouna and Ntoula (0.79 mosquitoes per house per night [m/h/n] and 0.49 m/h/n) for *An. gambiae* s.s.. The resting density of *An. coluzzii* was 0.16 m/h/n in Djoumouna and 0.20 m/h/n in Ntoula. The lowest resting density was recorded in *An. arabiensis* (0.02 m/h/n in Djoumouna and 0.03 m/h/n in Ntoula). These species of *An. gambiae* s.l. had higher resting density, with 0.26 m/h/n (8.58 mosquitoes per house per month [m/h/m]) in Ntoula, versus Djoumouna, where the resting density was 0.19 m/h/n (5.7 m/h/m) (Table 3).

### Anopheline man-biting rate

In Djoumouna village, persons received an average of 0.35 bites from *An. gambiae s.s.* per night, while 0.20 bites from *An. gambiae s.s.* per person per night were recorded in Ntoula village. *Anopheles coluzzii* had a man-biting rate of 0.10 bites per night in Ntoula compared with 0.07 bites per night in Djoumouna. *Anopheles arabiensis* was found to be less aggressive, with 0.01 bites per night in Djoumouna and 0.02 bites per night in Ntoula villages (Table 3).

### Entomological inoculation rate

The annual entomological inoculation rate was 37.4 infectious bites per person per year (ib/p/y) in all study sites. This entomological inoculation rate of the endophilic vector population was higher in Djoumouna (0.125 infectious bites per person per night [ib/p/n] or 45.6 ib/p/y) than in Ntoula (0.08 ib/p/n or 29.2 ib/p/y). The biting rates of *An. gambiae* s.s. were 0.11 ib/p/n (or 40.2 ib/p/y) and 0.04 ib/p/n (or 14.6 ib/p/y) in Djoumouna and Ntoula, respectively. The entomological inoculation rate was higher with *An. coluzzii* in Ntoula (0.03 ib/p/n or 11 ib/p/y) than in Djoumouna (0.01 ib/p/n or 3.7 ib/p/y). The lower entomological inoculation rates were recorded with *An. arabiensis* (0.005 ib/p/n and 0.01 ib/p/n in Djoumouna and Ntoula, respectively) (Table 3).

### Parity rate

Overall, out of 61 *An. gambiae* s.l. dissected, the parity and nulliparity rates were 52.5% (32/61) and 47.5% (29/61), respectively, in the Goma Tsé-Tsé district. A higher parity rate was recorded at Djoumouna (77.0%) than at Ntoula (23.0%) (*P* < 0.0001, OR = 11.7, 95% CI 4.83–25.23) (Table 3). In Djoumouna, the parity rate in *An. gambiae* s.s. was 85.7%, while that of *An. coluzzii* was 50%. Only *An. coluzzii* was parous in Ntoula, with a 75% parity rate (Table 3).

## Discussion

This study provides baseline entomological data on the composition of vector species involved in malaria transmission in the rural areas of Djoumouna and Ntoula (Republic of the Congo) during the dry season. *Anopheles gambiae* s.l. was the predominant mosquito species and the only anopheline mosquito found in the study area, and has been implicated in malaria transmission in Africa [16, 28]. The predominance of *An. gambiae* s.l. could be explained by the fact that they are ubiquitous species able to colonise several biotopes [29]. In fact, temporary puddles, residual pools of stagnant sunny surfaces, and ponds with erect vegetation have been found in these areas [30]. Previous studies carried out in the Republic of the Congo have also reported *An. gambiae* s.l. as a major malaria vector in savannah, forest, rural and urban areas [31–33].

*Anopheles gambiae* s.l. was more abundant in Djoumouna than in Ntoula, as compared with other mosquitoes. Indeed, Djoumouna village is surrounded by the presence of several permanent breeding sites, where secondary forest, farming activity and urbanisation, rainwater reservoirs and a combination of puddles, lakes, rivers, swamps and vegetable crops favour the development of *Anopheles* [31]. Ntoula is bordered by clean waterways such as the Congo River and various other rivers, which, in addition to the vegetable crops, favour the proliferation of *Anopheles* mosquitoes. In addition, according to the Simpson index in this study, the probability of encountering *An. gambiae* s.l. is significantly higher in Djoumouna than in Ntoula. Environmental changes induced by urbanisation (i.e. higher temperatures and lower relative humidity) could also provide favourable environments for the development of *An. gambiae* s.l. larvae in Djoumouna [5, 32].

This study is the first to report the different species of *An. gambiae* s.l. complex in rural areas of Djoumouna and Ntoula in the Republic of the Congo, since previous studies carried out in this setting used only morphological analysis for the identification of *An. gambiae* s.l. complex [34, 35]. Very few *An. arabiensis* specimens (4/144) were found in both villages, likely because anthropogenic actions such as deforestation and urbanization have destroyed its natural habitats, but this was not investigated herein. *Anopheles arabiensis* found in this study was a more exophilic and exophagic species. This behaviour was also reported in previous studies from Gambia [16, 34], Burkina-Faso [11]**,** Benin [29] and Kenya [36].

Only *P. falciparum*, *P. ovale* and *P. malariae* were found in *An. gambiae* s.l. Our observations differ from the findings reported by Carnevale et al. [18] showing only *P. falciparum* in the Republic of the Congo. *Plasmodium falciparum* was the most prevalent species in *An. gambiae* s.l. mosquitoes, followed by *P. malariae* and *P. ovale*, which were found in mono- or co-infection with *P. falciparum* in *An. coluzzii*, *An. gambiae* s.s. and *An. arabiensis*, and this explains its high prevalence previously reported in humans from the same area [19, 37].

*Anopheles coluzzii* was infected by mono-infection with *P. falciparum* or *P. malariae*. These results differ from the observations in Côte d'Ivoire [12], where *P. falciparum*, *P. malariae* and *P. ovale* were found in *An. coluzzii* and *P. falciparum* and *P. ovale* in *An. gambiae* s.s. The presence of *P. malariae* and *P. ovale* infections in *An. gambiae* s.l. in the present study is consistent with previous reports [12] and indicates the need for a national malaria control program to consider these two* Plasmodium* spp. when designing future measures for effective control and malaria treatment. As a limitation of the study, *Plasmodium* spp. detection was performed by PCR from the DNA extracted from the whole mosquitoes and not from the head–thorax only, which thus may have overestimated the indexes of infection rate. In addition, the origin of the *Anopheles* spp. blood meal was not investigated.

The cycle of aggression of *An. gambiae* s.l. shows that Djoumouna inhabitants received more bites per night than those of Ntoula. *Anopheles gambiae* s.s. was more aggressive than *An. coluzzi* and *An. arabiensis* in both villages. The biting activity of *Anopheles gambiae* s.s. was slightly higher in Djoumouna than in Ntoula, but the opposite was shown in *An. coluzzii* and *An. arabiensis* in Ntoula. This study also showed possible malaria transmission by different mosquito species in the study area, as previously found in several sites in Central African [33, 41] and West African countries [33, 39]. Overall, the estimated entomological inoculation rate was 37.4 ib/p/y. This rate is higher than that observed by Trape and Zoulani [30] (22.5 ib/p/y) in Brazzaville in 1987. This can be explained by the fact that Ntoula and Djoumouna belong to rural areas known to be associated with high malaria transmission [33, 42].

Ovarian dissection was performed to determine the parity status of *An. gambiae* s.l. females. The parturition rate observed in this study indicates an older population of *An. gambiae* s.l., which is associated with increased vectorial capacity as reported previously [11]. Most *An. gambiae* s.l. females caught in Djoumouna were parous, whereas those caught in Ntoula were mostly nulliparous. *Anopheles gambiae* s.s. caught at Ntoula and *An. arabiensis* from both Ntoula and Djoumouna villages were nulliparous, although the infection rates with *Plasmodium* species in these two *Anopheles* spp. were 0.19 and 0.5, respectively. This can be explained by the small sample size of mosquitoes included in the parity investigation test. Indeed, the very limited sample size (only 176 mosquitoes) is a major limitation of the present study, so all data analysis results reported herein should be interpreted with caution.

## Conclusions

This study provides baseline information on the dominant vectors and dynamics of malaria transmission in rural areas in the Republic of the Congo during the dry season. In the two sampled villages, *An. gambiae* complex mosquitoes, and *An. gambiae *s.s. in particular, play a predominant role in transmitting multiple *Plasmodium* species in the region. These findings highlight the need for improved vector control strategies and continuous monitoring of mosquito vectors to effectively combat malaria in the area.

### Supplementary Information

**Additional file 1: Table S1**: Primers used in the molecular identification of *An. gambiae* s.l. and the detection of *Plasmodium* spp.

## Data Availability

All data are fully available without restriction. Data are available from the Fondation Congolaise pour la Recherche Medical (FCRM) Institutional Data Access. All requests for data should be addressed to the Executive Director of FCRM, reachable at the following address: Prof. Francine Ntoumi, Villa D6, Cité OMS-Djoué, Brazzaville, République du Congo (Tel. +242-06-9977980, email: francine.ntoumi@uni-tuebingen.de.

## Abbreviations

CeRMI: Centre de recherche sur les maladies infectieuses-Christoph Merieux
LBDEA: Laboratoire de la Biodiversité et Ecologie Animales
FST: Faculté des Sciences et Techniques
UMNG: Université Marien Ngouabi
