# Supplementary material for: Contribution of Anopheles gambiae sensu lato mosquitoes to malaria transmission during the dry season in Djoumouna and Ntoula villages in the Republic of the Congo
Source: Parasit Vectors. 2024 Mar 2;17:104. doi: 10.1186/s13071-023-06102-7 (PMC10908062; doi:10.1186/s13071-023-06102-7)
Supplement: Supplementary file 1 — Additional file 1: Table S1: Primers used in the molecular identification of An. gambiae s.l. and the detection of Plasmodium spp. [file 13071_2023_6102_MOESM1_ESM.docx]

**Table S1**: Primers using in molecular identification of *An. gambiae s.l.* and the detection of *Plasmodium spp*

|  | **Primer** | **Primer Sequences** | Attending bande |
| --- | --- | --- | --- |
| **Primers** | Plas_F | 5'-GCTTAGTTACGATTAATAGGAGTAGCTTG-3' |  |
|  | Plas_R | GAAAATCTAAGAATTTCACCTCTGACA-3' |  |
| **Probe** | Falci+ | 5'- TCTGAATACGAATGTC-3' | FAM |
|  | OVM+ | 5'- CTGAATACAAATGCC-3' | HEX |
| **First round PCR** | rPLU6 | 5’-TTA AAA TTG TTG CAG TTA AAA CG-3’ |  |
|  | rPLU5 | 5’-CCT GTT GTT GCC TTA AAC TTC-3’ |  |
| **Second round PCR** | rFAL1 | 5’-TTA AAC TGG TTT GGG AAA ACC AAA TAT ATT-3’ | 205 bp |
|  | rFAL2 | 5’-ACA CAA TGA ACT CAA TCA TGA CTA CCC GTC-3’ |  |
|  | rMAL1 | 5’-ATA ACA TAG TTG TAC GTT AAG AAT AAC CGC-3’ | 105 bp |
|  | rMAL2 | 5’-AAA ATT CCC ATG CAT AAA AAA TTA TAC AAA-3’ |  |
|  | rOVA1 | 5’-ATC TCT TTT GCT ATT TTT TAG TAT TGG AGA-3’ | 800 bp |
|  | rOVA2 | 5’-GGA AAA GGA CAC ATT AAT TGT ATC CTA GTG-3’ |  |
|  | rVAV1 | 5’-GCT TCG GCT TGG AAG TCC-3' | 120 bp |
|  | rVAV2 | 5’-CCG AAT TCA GTC CCA CGT-3' |  |
| ***SINE200*** | Sine_F | TCG CCT TAG ACC TTG CGT TA | Ag ss= 249 bp  Ac= 479, Aa= 223 |
|  | Sine_R | CGC TTC AAG AAT TCG AGA TAC |  |

*Ag ss : An. gambiae ss; Ac : An. coluzzii* and *Aa : An. arabiensis,*
